# Phenotype and genotype of autosomal dominant tubulointerstitial kidney disease in a Japanese cohort

**DOI:** 10.1007/s10157-025-02629-4

**Published:** 2025-02-20

**Authors:** Yu Tanaka, China Nagano, Nana Sakakibara, Eri Okada, Shuhei Aoyama, Yuka Kimura, Yuta Inoki, Yuta Ichikawa, Chika Ueda, Hideaki Kitakado, Tomoko Horinouchi, Tomohiko Yamamura, Shingo Ishimori, Kazumoto Iijima, Kandai Nozu, Naoya Morisada

**Affiliations:** 1 Department of Pediatrics, Kobe University Graduate School of Medicine, 7-5-1, Kusunoki-Cho, Chuo-Ku, Kobe, Hyogo 650-0017 Japan; 2 Department of Clinical Genetics, Hyogo Prefectural Kobe Children’s Hospital, 1-6-7, Minatojimaminami-Machi, Chuo-Ku, Kobe, Hyogo 650-0047 Japan; 3 Hyogo Prefectural Kobe Children’s Hospital, 1-6-7, Minatojimaminami-Machi, Chuo-Ku, Kobe, Hyogo 650-0047 Japan

**Keywords:** Autosomal dominant tubulointerstitial kidney disease, *UMOD*, *MUC1*, *REN*, *SEC61A1*, Chronic kidney disease

## Abstract

**Background:**

Autosomal dominant tubulointerstitial kidney disease (ADTKD) is characterized by tubular atrophy, interstitial fibrosis, and progressive kidney dysfunction. Its causative genes include *UMOD, MUC1, REN, HNF1B*, and *SEC61A1*. ADTKD contributes to unexplained chronic kidney disease (CKD), and many cases remain genetically undiagnosed. This study aimed to elucidate the clinical features of patients genetically diagnosed with ADTKD in Japan.

**Methods:**

We included individuals with suspected congenital anomalies of the kidney and urinary tract, nephronophthisis, polycystic kidney disease, or ADTKD. Genetic analyses using direct sequencing, short-read next-generation sequencing (SRS), and/or long-read next-generation sequencing (LRS) were performed on 1097 families. Patients with ADTKD–*HNF1B* were excluded due to prior reporting.

**Results:**

Variants in *UMOD, MUC1, REN,* and *SEC61A1* were identified in 52 patients from 40 families (18, 16, 5, and 1 family, respectively). The median age at diagnosis was 38.5 years, and the urinary protein-to-creatinine ratio was 0.05 g/gCr. End-stage kidney disease was present at diagnosis in 37% of patients. Genetic testing was performed in 58% due to suspected ADTKD based on pathology or clinical course and in 38% due to unexplained CKD. Kidney biopsies were performed in 55%, with ADTKD confirmed pathologically in 41%. SRS and LRS were used in 55% and 30% of all families, respectively; for ADTKD–*MUC1,* 75% of families were analyzed using LRS.

**Conclusions:**

Clinical and pathological diagnosis of ADTKD remains challenging, emphasizing the importance of comprehensive genetic testing. Enhanced access to advanced genetic testing such as LRS is essential to improve diagnostic precision and management.

**Supplementary Information:**

The online version contains supplementary material available at 10.1007/s10157-025-02629-4.

## Introduction

Autosomal dominant tubulointerstitial kidney disease (ADTKD) is a common monogenic kidney disease characterized by tubular atrophy, interstitial fibrosis, and progressive kidney dysfunction [1]. A definitive diagnosis of ADTKD is made when an autosomal dominant family history of chronic kidney disease (CKD) presents these clinical and pathological features or when a causative gene is identified in an affected family member [2]. The main causative genes of ADTKD are *UMOD* [3], *MUC1* [4], *REN* [5], *HNF1B* [6], and *SEC61A1* [7]. ADTKD–*REN* has only been reported in 30 families [8] and ADTKD–*SEC61A1* in a few families [9].

Different from other ADTKDs, ADTKD–*HNF1B* or *HNF1B* nephropathy presents cystic kidney disease, congenital anomalies of the kidney and urinary tract (CAKUT), and tubular dysfunction. We previously reported the clinical features of 33 ADTKD–*HNF1B* cases at our institution in a Japanese population [6]. However, comprehensive reports on other types of ADTKD in a Japanese population are lacking. Therefore, this study aims to elucidate the genetic and clinical characteristics of patients with ADTKD–*UMOD*, ADTKD–*MUC1*, ADTKD–*REN*, and ADTKD–*SEC61A1* in Japan.

## Methods

### Patients

We conducted genetic testing on 1097 families of patients with CKD featuring minimal urinary abnormalities between 2010 and August 2023. The patients from across Japan were suspected to have CAKUT, nephronophthisis (NPHP), polycystic kidney disease, and ADTKD. Aside from variant data, we reviewed clinical information on extrarenal manifestations, renal findings, onset age, and laboratory data. Clinical information was retrospectively reviewed from medical records provided by clinicians during genetic testing. All samples and clinical information were obtained from Japanese referral hospitals. Hyperuricemia was defined as serum uric acid concentrations > 7.0 mg/dL. This study was approved by the Institutional Review Board of Kobe University School of Medicine, and genetic analyses were performed after obtaining informed consent (approval numbers 86, 301, and 1210).

### Genetic analysis

Genomic DNA was isolated from peripheral blood leukocytes of the patients and their families using the QuickGene whole blood kit S (Kurabo, Osaka, Japan). Direct or targeted short-read next-generation sequencing (SRS) was conducted using HaloPlex HS or SureSelect (Agilent Technologies, Santa Clara, CA, USA). Paired-end sequencing was performed on the MiSeq platform (Illumina, San Diego, CA, USA). HaloPlex HS and SureSelect were used to sequence genes associated with CAKUT, NPHP-related ciliopathies, polycystic kidney disease, and ADTKD, as cataloged in the OMIM database (http://www.omim.org/) or PubMed (http://www.ncbi.nlm.nih.gov/pubmed). *DNAJB11,* a recently identified causative gene for ADTKD, was included in versions 7–12 of SureSelect panels (Supplementary Table 3). SRS samples were prepared following the manufacturer’s instructions. Briefly, for HaloPlex HS, 225 ng genomic DNA was restriction digested and hybridized at 54 °C for 16 h using next-generation sequencing (NGS) probes. For SureSelect, 225 ng genomic DNA was restriction digested and hybridized at 65 °C for 24 h following the manufacturer’s protocol. All indexed and captured DNA samples were amplified through polymerase chain reaction (PCR) and sequenced using the MiSeq platform (Illumina). SRS data were analyzed using SureCall software v4.2 (Agilent Technologies). Variants detected through SRS were confirmed by direct Sanger sequencing using a 3130 Genetic Analyzer (Thermo Fisher Scientific, Tokyo, Japan) and SeqStudio Genetic Analyzer (Thermo Fisher Scientific). In patients clinically suspected of having ADTKD–*UMOD* or ADTKD–*REN*, Sanger sequencing was first performed for all exons of *UMOD* and exon 1 of *REN* prior to SRS.

In patients clinically suspected with ADTKD for whom no pathogenic variants were detected by SRS, long-read NGS (LRS) was subsequently performed. Suspected ADTKD before genetic testing was defined based on the presence of clinical features of ADTKD, along with either a family history of autosomal dominant inheritance or pathological findings suggestive of ADTKD. PCR primers were designed to amplify the variable number of tandem repeat (VNTR) region of *MUC1* for LRS using PacBio barcodes for SMRT sequencing (Pacific Biosciences, Menlo Park, CA, USA). This sequencing was performed on pooled amplicons, and the "pbaa" tool was used to cluster repetitive sequences. Correct sequences for each patient were identified by comparing SMRT sequencing results with electropherogram data, accounting for potential PCR errors in the VNTR region. Details of the *MUC1* analysis method using LRS were previously reported [10]. Direct sequencing data were analyzed using CLC Main Workbench version 6.7.1 (CLC bio, Aarhus, Denmark). We used gnomAD (https://gnomad.broadinstitute.org/) to reference allele frequencies and consulted ClinVar (https://www.ncbi.nlm.nih.gov/clinvar/) and the Human Gene Mutation Database (https://www.hgmd.cf.ac.uk) as variant databases. Candidate variants were selected using CADD (https://cadd.gs.washington.edu/snv), PolyPhen-2 (http://genetics.bwh.harvard.edu/pph2/), and MutationTaster (http://www.mutationtaster.org/) and then classified as pathogenic, likely pathogenic, or uncertain significance, according to the guidelines of the American College of Medical Genetics (ACMG).

### Statistical analysis

Data are reported as median and interquartile range. Continuous variables were analyzed using the Mann–Whitney rank-sum test or unpaired *t* test. Nominal variables were analyzed using Fisher’s exact test. *P* < 0.05 was considered to indicate statistical significance. All statistical analyses were performed using Easy R (EZR) [11]. Kaplan–Meier curves were generated for the time taken to develop end-stage kidney disease (ESKD).

## Results

### All patients

**Fig. 1:**
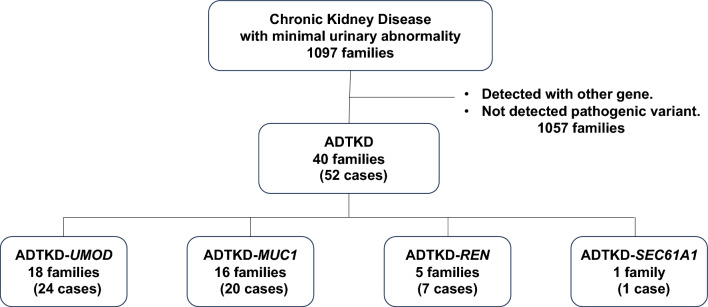
Patients in this cohort

In total, 52 patients from 40 families were genetically diagnosed with ADTKD from 1097 families who had CKD with minimal urinary abnormalities. ADTKD–*UMOD* was identified in 24 patients (18 families), ADTKD–*MUC1* in 20 patients (16 families), ADTKD–*REN* in 7 patients (5 families), and ADTKD*–SEC61A1* in 1 patient (1 family) (Fig. 1, Supplementary Table 1). The median diagnostic age was 38.5 years, and ESKD was present at diagnosis in 37% (18/49) of the patients (Table 1). Of the patients genetically diagnosed with ADTKD, 58% (23/40) were clinically suspected of ADTKD prior to genetic analysis. In addition, 38% (15/40) of the patients underwent genetic testing due to unexplained CKD. Kidney biopsies were performed in 55% (22/40), with histopathological diagnoses including ADTKD in 41% (9/22) and NPHP or ADTKD in 14% (3/22) (Supplementary Table 2). In addition, in 23% (9/40), genetic testing was prioritized due to a clinical suspicion of ADTKD (Supplementary Table 1).

**Table 1:** Clinical characteristics of all cases diagnosed with ADTKD

	ADTKD 52cases
Age at diagnosis (year)	38.5 (22.5–48.3)
Sex female (%)	38 (20/52)
Urine protein-to-creatinine ratio (g/gCr)	0.05 (0.01–0.13)
Systolic blood pressure (mmHg)	124.5 (113.0–135.3)
Cr–eGFR (mL/min/1.73m^2^)	37.8 (20.5–53.6)
ESKD (%)	37 (18/49)

Cr–eGFR: creatinine-based estimated glomerular filtration rate, ESKD: end stage kidney disease

### ADTKD–*UMOD*

The median age at diagnosis was 30.5 years, and creatinine-based estimated glomerular filtration rate (Cr–eGFR) was 37.4 mL/min/1.73 m^2^, with ESKD present in 30% (7/23) of the patients. The median serum uric acid level was 7.4 mg/dL, with 94% (15/16) of the patients having hyperuricemia or receiving treatment. Missense and indel variants were found in 94% (17/18) and 6% (1/18) of the families, respectively. Of the 18 variants, 94% (17/18) were found in exon 3, with missense variants in the cysteine residue accounting for 50% (9/18). Two variants were classified as variants of uncertain significance (VUSs): c.746_763del (SC902) and c.483C > G (SC967). Despite having a family history suggestive of autosomal dominant inheritance, segregation analysis was not performed. Neither variant was listed in gnomAD, and c.483C > G was predicted to be pathogenic by in silico analyses. Both cases exhibited early progressing kidney dysfunction and hyperuricemia (Table 2, Supplementary Table 1).

**Table 2:** Clinical characteristics of ADTKD–*UMOD* and ADTKD–*MUC1*

	*UMOD* 24 (18)	*MUC1* 20 (16)	*P* value
Sex female (%)	33 (8/24)	50 (10/20)	0.36
Familial history of CKD or Gout (%)	78 (14/18)	88 (14/16)	0.66
Age at diagnosis (year)	30.5 (20.3–53.0)	41.0 (37.5–47.0)	0.46
Child under 18 years (%)	21 (5/24)	5 (1/20)	0.20
Systolic blood pressure (mmHg)	124.5 (118.0–137.0)	127.5 (118.8–131.5)	0.94
Cr–eGFR (mL/min/1.73m^2^)	37.4 (28.0–53.9)	23.0 (9.9.–41.0)	0.38
ESKD (%)	30 (7/23)	40 (8/20)	0.54
Urine protein-to-creatinine ratio (g/gCr)	0.04 (0.00–0.09)	0.11 (0.01–0.13)	0.44
Serum uric acid (mg/dL)	7.4 (6.1–8.4)	6.3 (5.9–6.9)	0.06
Hyperuricemia or treatment (%)	94 (15/16)	63 (10/16)	0.08

CKD: chronic kidney disease, Cr–eGFR: creatinine-based estimated glomerular filtration rate, ESKD: end stage kidney disease

### ADTKD–*MUC1*

The median age at diagnosis was 41 years, and Cr–eGFR was 23.0 mL/min/1.73 m^2^, with ESKD present in 40% (8/20) of the patients. The median serum uric acid level was 6.3 mg/dL, with 63% (10/16) of the patients having hyperuricemia or receiving treatment. SRS and LRS were used in 25% (4/16) and 75% (12/16) of the patients, respectively. All patients had a frameshift variant due to a single base insertion. Patients diagnosed using SRS had variants either within the third repeat of the VNTR or in the region upstream of the VNTR (Table 2, Supplementary Table 1).

### Comparison of clinical features between ADTKD–*UMOD* and ADTKD–*MUC1*

**Fig. 2:**
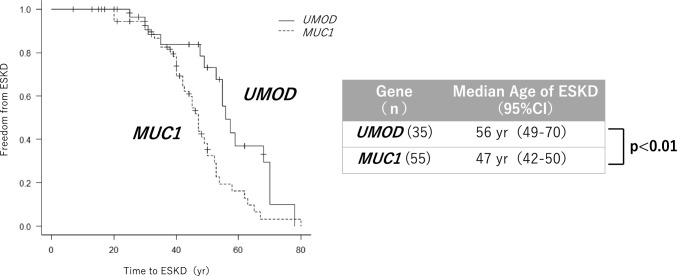
Kaplan–Meier curves of ESKD onset age for ADTKD–*UMOD* and ADTKD–*MUC1*

No significant differences in almost all of characteristics were observed between ADTKD–*UMOD* and ADTKD*–MUC1* (Table 2). Kaplan–Meier analysis of the age at ESKD onset, including familial CKD cases, revealed a median ESKD onset age of 56 years (95% CI 49–70) for ADTKD*–UMOD* and 47 years (95% CI 42–50) for ADTKD*–MUC1*, with ADTKD–*MUC1* showing a significantly younger onset age (Fig. 2).

### ADTKD–*REN*

**Fig. 3:**
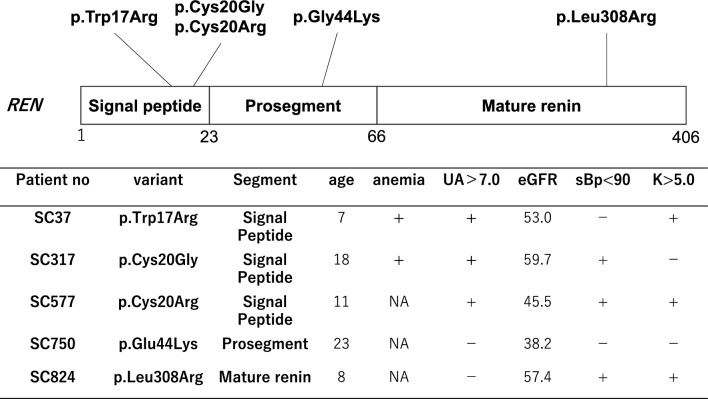
Phenotypic differences in ADTKD–*REN* based on genetic variants

The median age was 14.5 years. Pediatric diagnoses accounted for 57% (4/7). A family history was observed in 60% (3/5) of the families. The median systolic blood pressure was 87.0 mmHg, and the Cr–eGFR was 53.0 mL/min/1.73 m^2^, with no cases of ESKD (0/7). The median serum uric acid level was 7.3 mg/dL, with 100% (7/7) of the patients having hyperuricemia or receiving treatment. Hemoglobin levels were 9.0 g/dL, and hyperkalemia was observed in 60% (3/5) of the patients. All patients had missense variants, with 80% (4/5) found in exons 1 and 2 (Fig. 3, Table 3, Supplementary Table 1). The SC824 case had a variant in the mature renin region of *REN* and exhibited diverse symptoms. Impaired kidney function was observed at the age of 5, while extrarenal symptoms, including motor developmental delay with hypotonia, exotropia, cryptorchidism, short neck, and low set ears, were noted from infancy. The cause of these extrarenal symptoms remained unknown, and *CHD7* variant, the causative gene for CHARGE syndrome, was not detected. Family history revealed multiple cases of CKD on the paternal side, but no extrarenal symptoms similar to those observed in SC824 were reported.

**Table 3:** Clinical characteristics of ADTKD–*REN*

	*REN-*7(5)
Sex female (%)	25 (2/8)
Familial history of CKD or Gout (%)	60 (3/5)
Age (years)	14.5 (8.8–21.8)
Child under 18 years (%)	57 (4/7)
Systolic blood pressure (mmHg)	87.0 (86.0–100.0)
Cr–eGFR (mL/min/m^2^)	53.0 (45.5–57.4)
ESKD (%)	0
Urine protein-to-creatinine ratio (g/gCr)	0.09 (0.06–0.10)
Serum uric acid (mg/dL)	7.3 (6.0–7.4)
Hyperuricemia or treatment (%)	100 (7/7)
Hemoglobin (g/dL)	9.0 (8.8–9.3)
Hyperkalemia (%)	60 (3/5)

CKD: chronic kidney disease, Cr–eGFR: creatinine-based estimated glomerular filtration rate, ESKD: end stage kidney disease

### ADTKD–*SEC61A1*

The patient was a 26-year-old male with no family history of CKD. He presented with normocytic anemia, kidney dysfunction, renal tubular acidosis, and mild hypotension during infancy. The systolic blood pressure was 99 mmHg, and the Cr–eGFR was 61.8 mL/min/1.73 m^2^. The serum uric acid level was 6.3 mg/dL under medication. The urinary protein-to-creatinine ratio was 0.21 g/gCr. A novel missense variant that is extremely rare and was predicted to have high pathogenicity based on in silico analysis was detected using SRS (Supplementary Table 1).

## Discussion

ADTKD often leads to CKD without diagnosis owing to minimal urinary abnormalities and is a major monogenic kidney disease in unexplained CKD cases. Diagnosing ADTKD based on clinical symptoms and pathology is challenging, making comprehensive genetic testing crucial. International cohort studies have reported that 9% of adult patients with CKD have hereditary kidney disease, with *UMOD* accounting for 3% [12]. Genetic testing of ESKD patients with a family history revealed 0.5% were diagnosed with ADTKD [13], highlighting ADTKD as a common monogenic cause of unexplained CKD [14]. In this study, 33% of the patients genetically diagnosed with ADTKD had initially presented with unexplained CKD. In addition, 59% were not pathologically diagnosed with ADTKD after kidney biopsy. Clinical and pathological diagnosis of ADTKD remains challenging, highlighting the renewed importance of genetic testing.

The clinical and genetic features of ADTKD–*UMOD* in this study were consistent with those in previous reports. In an international cohort including 586 suspected ADTKD families, ADTKD*–UMOD* accounted for 38.4% of the cases [15]. In this study, it represented 46%. A previous report indicated a median diagnosis age of 42 years, eGFR of 39.2 mL/min/1.73 m^2^, and ESKD prevalence of 44% [15]. Compared with the patients in this study, these patients were later diagnosed with more advanced kidney dysfunction, suggesting an earlier diagnosis in Japan. ADTKD–*UMOD* is characterized by early onset gout due to reduced NaK2Cl channel activation, leading to decreased extracellular fluid and increased uric acid reabsorption [9]. Although detailed gout data were not collected, hyperuricemia was found in 93% of our patients, consistent with 87% in a report of 44 patients with ADTKD–*UMOD* [16]. Almost none of the patients in this study exhibited proteinuria, and only one (SC1037) presented with heavy proteinuria. The incidence of mild proteinuria is estimated to be 10%, while severe proteinuria is considered rare [16]. In a previous study, 95.3% of ADTKD–*UMOD* variants are missense, 4.7% indels, with 53.8% of missense variants affecting cysteine residues [15]. Similarly, 94% (17/18) of the variants in this study were missense, 6% (1/18) indels, 50% (9/18) affected cysteine residues, and 94% (17/18) were in exon 3. Thus, the clinical and genetic features of ADTKD*–UMOD* in Japan are similar to those previously reported. However, in this study, SC902 and SC967 had variants still classified as VUS, and additional analyses are required to confirm their pathogenicity.

ADTKD–*MUC1*, the second most common causative gene [15], accounts for 35% of cases, which is consistent with our findings. It follows an autosomal dominant pattern and causes kidney dysfunction in adulthood without prominent clinical features. Genetic testing is essential for diagnosis, but *MUC1* analysis is challenging, because its VNTR region consists of 60 base pairs repeated 25–120 times. ADTKD*–MUC1* is caused by a frameshift variant resulting from a single base insertion in the VNTR region. Accumulation of the resulting MUC1-fs causes tubular atrophy, apoptosis, and kidney impairment [17]. Conventional SRS cannot analyze the VNTR region, which complicates genetic testing. In this study, we detected pathogenic variants in 16 families (20 patients) using either SRS or LRS. We performed LRS in cases in which variants were not detected using SRS. SRS was used to analyze 25% (4/16) of cases, but 75% (12/16) required LRS. SRS can only analyze up to the third VNTR repeat, with more distal units requiring LRS for accurate diagnosis.

The technical challenges and high costs of *MUC1* analysis limit access to genetic testing, which is crucial for accurate diagnosis. Consequently, many undiagnosed ADTKD*–MUC1* cases may be misclassified as ADTKD–NOS, with additional undiagnosed cases likely hidden. Approximately 60% of patients with clinically diagnosed ADTKD receive a confirmed genetic diagnosis, leaving many patients without an identified causative gene [9]. Among these patients, some could also be undiagnosed with ADTKD–*MUC1*. Various diagnostic methods, including mass spectrometry, SMRT, SNaPshot, MUC1-fs staining, and alignment-free variant calling, have been explored [4, 10, 18–21], but most are not widely available. We also developed multiple *MUC1* analysis methods and hope that these advancements will increase ADTKD–*MUC1* cases and improve patient outcomes.

We compared the clinical features of ADTKD–*UMOD* and ADTKD–*MUC1*. These genes account for approximately 70% of clinically diagnosed ADTKD cases [15], but distinguishing them clinically is challenging. In this study, the age of ESKD onset was significantly younger in ADTKD–*MUC1* than in ADTKD–*UMOD* (47 vs. 56 years, *P* < 0.01), consistent with previous reports (46 vs. 54 years, *P* = 0.013) [15]. Similarly, while hyperuricemia was more prevalent in ADTKD*–UMOD* than in ADTKD–*MUC1* in this study, this difference was not statistically significant (94% vs. 63%, *P* = 0.06). Conversely, prior studies reported significant differences in the prevalence rates of ADTKD–*UMOD* and ADTKD–*MUC1* (87% vs. 54%, *P* < 0.01). The age at diagnosis was also consistent with previous reports, with ADTKD–*UMOD* diagnosed earlier than ADTKD–*MUC1* (30.5 vs. 41.0 years in this study, 32.4 vs. 40.8 years in a previous report). Although this difference was not significant in this study (P = 0.46), prior reports demonstrated a statistically significant earlier diagnosis in ADTKD–*UMOD* (P = 0.03) [16]. Although this study did not assess the age of onset for gout, prior research has shown that gout occurs significantly earlier in ADTKD–*UMOD* than in ADTKD*–MUC1* [15], suggesting that early onset gout may serve as a useful clinical marker for distinguishing these two conditions. No significant differences in other clinical characteristics were observed in either this study or previous reports, highlighting the challenges of distinguishing ADTKD–*UMOD* and ADTKD–*MUC1* based solely on clinical features [15, 16]. For cases resembling ADTKD–*UMOD* but with negative NGS results, ADTKD–*MUC1* should be considered, and specialized genetic testing may be necessary to ensure accurate diagnosis.

ADTKD–*REN* is characterized by various extrarenal symptoms, with many diagnosed during childhood*.* It can be divided into three types: signal peptide variants, where abnormal preprorenin fails to translocate into the ER; mature renin variants, where abnormal prorenin accumulates in the ER; and prosegment variants, where prorenin is deposited in the ER–Golgi intermediate compartment, affecting protein synthesis [8]. These pathological differences influence the phenotypes [9]. ADTKD–*REN* involves CKD with reduced renin production, leading to mild hypotension, hyperkalemia, childhood anemia, hyperuricemia, and recurrent acute kidney injury (AKI) [5, 22]. In the present study, three of five patients had signal peptide variants, consistent with 62% in an international cohort [8]. These variants are often severe, with two of the three patients presenting with anemia, mild hypotension, and hyperkalemia and all presenting with hyperuricemia. SC824 was found to have a variant in the mature region. Previous reports showed that variants in this region were characterized by the latest onset of kidney dysfunction and an absence of extrarenal symptoms other than gout [8]. This case exhibited only mild hyperkalemia without any history of anemia, acidosis, or acute kidney injury. In addition, multiple cases of kidney dysfunction were identified in the paternal family, but none presented with the extrarenal symptoms observed in SC824. Thus, *REN* variants are likely associated with kidney phenotype but not with extrarenal manifestations. Moreover, although this variant is novel, it is considered a pathogenic variant due to its extreme rarity, the high pathogenicity indicated by in silico analyses, and its detection in the father, who also presented with CKD. Thus, it is strongly suspected to be a pathogenic variant. Unlike other ADTKD types, ADTKD–*REN* presents childhood symptoms due to reduced renin production, with four out of five cases diagnosed before age 20, confirming its childhood onset.

ADTKD–*SEC61A1* is extremely rare, with only five families reported [7, 12, 23]. Its phenotypes include intrauterine growth retardation, growth disorders, cleft palate, polydactyly, velopharyngeal insufficiency, congenital anemia, leukopenia, mild motor development delay, and polyuria, with significant clinical variability [7, 23]. Our patient had no family history of kidney disease but was diagnosed with normocytic anemia, kidney dysfunction, and type 4 distal renal tubular acidosis at 11 months and treated with fludrocortisone for mild hypotension. A kidney biopsy at 1 year revealed interstitial nephritis, and the patient experienced recurrent AKI due to mild dehydration. These features strongly suggested ADTKD–*REN*, and genetic testing identified a novel missense variant. In silico analyses (CADD, PROVEAN, SIFT, PolyPhen-2, MutationTaster) indicated pathogenicity, and the variant was not found in a healthy population database. In addition, variants in nearby residues of our variant have been previously reported [7]. When classified as a VUS according to the ACMG criteria, the phenotype matched that of ADTKD–*SEC61A1*, leading to a diagnosis. The *SEC61A1* variant affects the translocon, influencing protein folding and membrane translocation, and potentially affects uromodulin, mucin1, and renin, leading to symptoms caused by abnormal proteins [9]. This broad pathogenic impact explains clinical variability. However, functional analysis is required to establish the pathogenicity of this variant. Increasing genetic diagnoses may clarify genotype–phenotype relationships.

### Limitation

This study has some limitations. First, the data were collected from ADTKD cases across Japan, which may introduce variability in pathological and clinical findings between institutions. In addition, this study focused on cases suspected of polycystic kidney disease, CAKUT, ADTKD, and NPHP with limited urinary findings and did not include cases with nephritis or nephrotic syndrome, which may represent different clinical manifestations. Given the difficulty to clinically suspect ADTKD–*MUC1*, some patients may not have undergone LRS, potentially leading to its underdiagnosis. Moreover, the gene panels used for analysis were incrementally updated, leading to differences in the genes analyzed across versions. For ADTKD, *SEC61A1* was excluded in versions 2 and 4, while *DNAJB11* was excluded in versions 2, 4, 5, or 6. In this study, we rigorously evaluated both phenotypes and genotypes and included cases deemed pathogenic. However, some variants classified as VUS according to ACMG criteria were included, and further investigation into their pathogenicity is necessary.

## Conclusion

This study is the first to comprehensively describe the clinical features of genetically confirmed ADTKD in a Japanese population. A significant number of patients with unexplained CKD were included among the diagnosed patients in this study. The clinical diagnosis of ADTKD is challenging, and comprehensive genetic testing is essential for accurate diagnosis. Improving the diagnostic accuracy of ADTKD requires the establishment and widespread implementation of *MUC1* analysis methods in routine clinical practice.

## Supplementary Information

Below is the link to the electronic supplementary material.

Supplementary file1 (DOCX 29 KB) Supplementary Table 1: Overview of ADTKD variants in this cohort

Supplementary file2 (DOCX 31 KB) Supplementary Table 2: Overview of clinical characteristics of all patients in this cohort

Supplementary file3 (DOCX 56 KB) Supplementary Table 3: Gene panels for short read sequencing

Supplementary file4 (DOCX 16 KB) Supplementary Table 4: References of Reports in the study
