# Supplementary material for: Phenotype and genotype of autosomal dominant tubulointerstitial kidney disease in a Japanese cohort
Source: Clin Exp Nephrol. 2025 Feb 20;29(6):788–96. doi: 10.1007/s10157-025-02629-4 (PMC12125067; doi:10.1007/s10157-025-02629-4)
Supplement: Supplementary file 1 — Supplementary file1 (DOCX 29 KB) Supplementary Table 1: Overview of ADTKD variants in this cohort [file 10157_2025_2629_MOESM1_ESM.docx]

Supplementary Table 1

PD：Possibly Damaging, D: Damaging, B:Benign, NA: Not Applicable, VUS: Variant of Uncertain Significance, DM: Disease mutation, SRS: short-read next-generation sequencing, LRS: long-read next-generation sequencing
