# Supplementary material for: Phenotype and genotype of autosomal dominant tubulointerstitial kidney disease in a Japanese cohort
Source: Clin Exp Nephrol. 2025 Feb 20;29(6):788–96. doi: 10.1007/s10157-025-02629-4 (PMC12125067; doi:10.1007/s10157-025-02629-4)
Supplement: Supplementary file 2 — Supplementary file2 (DOCX 31 KB) Supplementary Table 2: Overview of clinical characteristics of all patients in this cohort [file 10157_2025_2629_MOESM2_ESM.docx]

| Supplementary Table 2 | |  |  |  |  |  |  |  |
| --- | --- | --- | --- | --- | --- | --- | --- | --- |
| Patient ID | Age at diagnosis | SEX | Cr-eGFR  (ml/min/1.73m^2^) | Suspected  diagnosis | Pathological  findings | CKD stage5 | Familial history | Extrarenal manifestation |
| ADTDK-*UMOD* | | | | | | | | |
| SC50 | 31 | F | 59.3 | CKD | Done (NA) | No | Yes | Deafness |
| SC50A | 56 | M | KRT |  |  | Yes | Yes | Gout |
| SC395 | 54 | M | KRT | CKD | Nephrosclerosis | Yes | No |  |
| SC408 | 30 | M | 36.4 | CKD | ADTKD | No | Yes |  |
| SC408B | 60 | F | NA |  |  | NA | Yes |  |
| SC430 | 17 | M | 53.9 | CKD | Not done | No | Yes |  |
| SC430B | NA | F | KRT |  |  | Yes | Yes |  |
| SC447 | 62 | M | 43.8 | ADTKD | MGA | No | Yes |  |
| SC492 | 50 | M | 35.6 | ADTKD | NPHP or ADTKD | No | Yes |  |
| SC660 | 44 | F | 37.3 | CKD | Not done | No | Yes |  |
| SC851 | 13 | F | 53.8 | CKD | Not done | No | No | Gall stones |
| SC872 | 49 | F | KRT | ADPKD | Not done | Yes | Yes |  |
| SC890 | 47 | M | 20 | ADTKD | Not done | No | Yes |  |
| SC902 | 30 | M | 37.4 | ADTKD | Not done | No | Yes |  |
| SC929 | 13 | M | 114.7 | ADTKD | Not done | No | Yes |  |
| SC929A | NA | M | KRT |  |  | Yes | Yes |  |
| SC966 | 29 | M | KRT | ADTKD | ADTKD | Yes | No |  |
| SC967 | 28 | F | 14.7 | ADTKD | Done (NA) | Yes | Yes | Congenital heart disease |
| SC1031 | 7 | F | 30 | NPHP | Not done | No | No | Convulsion |
| SC1037 | 68 | M | 22 | ADTKD | Not done | No | Yes | Diabetes type 2 |
| SC1061 | 21 | M | 16 | ADTKD | Done (NA) | No | Yes |  |
| SC1067 | 20 | M | 53.9 | ADTKD | Not done | No | Yes | Urinary stones |
| SC1067A | 62 | M | KRT |  |  | Yes | Yes |  |
| SC1067C | 16 | M | 65.8 |  |  | No | Yes |  |
| ADTKD-*MUC1* | | | | | | | | |
| SC265 | 43 | F | KRT | CKD | Done (NA) | Yes | Yes | Hyperkalemia |
| SC356 | 41 | F | 31.9 | ADTKD | NPHP or ADTKD | No | Yes |  |
| SC359 | 40 | M | 39.7 | ADTKD | ADTKD | No | Yes | Gout |
| SC370 | 15 | M | 110.8 | ADTKD | Done (NA) | No | Yes |  |
| SC416 | 32 | M | 23.0 | CKD | Nephritis | No | Yes |  |
| SC416C | 38 | F | 46.0 |  |  | No | Yes |  |
| SC416D | 35 | M | KRT |  |  | Yes | Yes |  |
| SC489 | 54 | M | KRT | ADTKD | Nephrosclerosis | Yes | Yes | Hyperkalemia |
| SC512 | 37 | M | 16.4 | ADTKD | ADTKD | No | No |  |
| SC534 | 40 | F | KRT | ADTKD | NPHP or ADTKD | Yes | Yes | Intellectual disability |
| SC534C | NA | M | KRT |  |  | Yes | Yes |  |
| SC566 | 50 | M | 10.0 | ADTKD | ADTKD | Yes | No |  |
| SC656 | 46 | F | 41.0 | CKD | Not done | No | Yes | Polycystic liver, Multiple colonic diverticula |
| SC696 | 48 | F | 9.9 | ADTKD | Not done | Yes | Yes | Hyperkalemia |
| SC732 | 68 | F | KRT | CKD | Not done | Yes | Yes |  |
| SC798 | 48 | F | KRT | ADTKD | MGA | Yes | Yes | Polycystic kidney |
| SC798C | 44 | F | 76.2 |  |  | No | Yes |  |
| SC870 | 25 | M | 24.5 | ADTKD | ADTKD | No | Yes |  |
| SC912 | 39 | F | 22.0 | ADTKD | ADTKD | No | Yes |  |
| SC995 | 46 | M | KRT | CKD | Not done | Yes | Yes |  |
| ADTKD-*REN* | | | | | | | | |
| SC37 | 7 | F | 53 | CKD | ADTKD | No | No | Hyperkalemia, Anemia |
| SC317 | 18 | F | 59.7 | CKD | Not done | No | Yes | Anemia |
| SC317A | 50 | M | NA |  |  | NA | Yes | Gout |
| SC577 | 11 | M | 45.5 | CKD | Not done | No | No | Hyperkalemia |
| SC750 | 23 | M | 38.2 | ADTKD | Not done | No | Yes | Low stature |
| SC824 | 8 | M | 57.4 | ADTKD | ADTKD | No | Yes | Hyperkalemia, Bilateral cryptorchidism, Exotropia, Psychomotor developmental delay, hypotonia, short neck with webbed neck, high-arched palate, low set ears |
| SC824A | NA | M | NA |  |  | NA | Yes |  |
| ADTKD-*SEC61A1* | | | | | | | | |
| SC778 | 26 | M | 61.8 | CKD | Not done | No | No | Epilepsy, Long QT syndrome |

F: Female, M: Male, KRT: Kidney replacement therapy, Cr-eGFR: Creatinine based estimated-glomerular filtration rate, CKD: chronic kidney disease
