# Supplementary material for: Phenotype and genotype of autosomal dominant tubulointerstitial kidney disease in a Japanese cohort
Source: Clin Exp Nephrol. 2025 Feb 20;29(6):788–96. doi: 10.1007/s10157-025-02629-4 (PMC12125067; doi:10.1007/s10157-025-02629-4)
Supplement: Supplementary file 3 — Supplementary file3 (DOCX 56 KB) Supplementary Table 3: Gene panels for short read sequencing [file 10157_2025_2629_MOESM3_ESM.docx]

Supplementary Table 3

Gene list of HaloPlex HS version 2

| *ACE* | *BMP7* | *CXCL12* | *FZD4* | *IQCB1* | *NPHS2* | *RET* | *TBX18* | *WNT4* |
| --- | --- | --- | --- | --- | --- | --- | --- | --- |
| *ACTN4* | *CC2D2A* | *CXCR4* | *FZD8* | *ITGA8* | *NUP107* | *ROBO2* | *TMEM138* | *WNT5A* |
| *ADCK4* | *CD2AP* | *DCDC2* | *GATA3* | *KAL1* | *NUP133* | *ROR1* | *TMEM231* | *WNT7A* |
| *AGT* | *CDC5L* | *DSTYK* | *GDNF* | *KIAA0586* | *NXF5* | *ROR2* | *TMEM237* | *WNT7B* |
| *AGTR1* | *CEP164* | *EP300* | *GLIS2* | *LAMB2* | *OFD1* | *RPGLIP1L* | *TMEM67* | *WNT9B* |
| *AGTR2* | *CEP290* | *EYA1* | *GREM1* | *LMNA* | *OSR1* | *SALL1* | *TNXB* | *WT1* |
| *AHI1* | *CEP41* | *FAT3* | *GRIP1* | *LMX1B* | *PAX2* | *SDCCAG8* | *TP53* | *ZEB2* |
| *ALG13* | *CEP83* | *FAT4* | *HNF1B* | *MDM2* | *PAX8* | *SIX1* | *TRAP1* | *ZNF423* |
| *ANKS3* | *CHD1L* | *FGF2* | *HOXA13* | *MKKS* | *PDE6D* | *SIX2* | *TRPC6* |  |
| *ANKS6* | *CHD4* | *FGF20* | *HOXD11* | *MUC1* | *PKD1* | *SIX5* | *TTC21B* |  |
| *ANLN* | *CHRM3* | *FGF9* | *IFN2* | *MYO1E* | *PKD2* | *SLIT2* | *UMOD* |  |
| *APOL1* | *CITED1* | *FOXD1* | *IFT172* | *NEK8* | *PKHD1* | *SOX17* | *UPK3A* |  |
| *ARL13B* | *COQ6* | *FRAS1* | *IFT81* | *NPHP1* | *PLCE1* | *SPRY1* | *VANGL2* |  |
| *BMP2* | *CSPP1* | *FREM1* | *INPP5E* | *NPHP3* | *PODXL* | *SRGAP1* | *WDR19* |  |
| *BMP4* | *CTDNEP1* | *FREM2* | *INVS* | *NPHP4* | *REN* | *TBX1* | *WNT11* |  |

Gene list of HaloPlex HS version 4

| *ACE* | *B9D1* | *CD2AP* | *CTDNEP1* | *FOXD1* | *IFN2* | *LMNA* | *NUP107* | *RET* | *TBX1* | *TSC1* | *WNT7B* |
| --- | --- | --- | --- | --- | --- | --- | --- | --- | --- | --- | --- |
| *ACTN4* | *B9D2* | *CDC5L* | *CXCL12* | *FRAS1* | *IFT27* | *LMX1B* | *NUP133* | *ROBO2* | *TBX18* | *TSC2* | *WNT9B* |
| *ADCK4* | *BBIP1* | *CENPF* | *CXCR4* | *FREM1* | *IFT43* | *LZTFL1* | *NXF5* | *ROR1* | *TCTN2* | *TTC8* | *WNT11* |
| *AGT* | *BBS1* | *CEP41* | *DCDC2* | *FREM2* | *IFT81* | *MDM2* | *OFD1* | *ROR2* | *TCTN3* | *TTC21B* | *WT1* |
| *AGTR1* | *BBS2* | *CEP83* | *DDX59* | *FZD4* | *IFT122* | *MKKS* | *OSR1* | *RPGLIP1L* | *TMEM67* | *UMOD* | *XPNPEP3* |
| *AGTR2* | *BBS4* | *CEP104* | *DSTYK* | *FZD8* | *IFT140* | *MKS1* | *PAX2* | *SALL1* | *TMEM138* | *UPK3A* | *ZEB2* |
| *AHI1* | *BBS5* | *CEP120* | *DYNC2H1* | *GANAB* | *IFT172* | *MUC1* | *PAX8* | *SDCCAG8* | *TMEM216* | *VANGL2* | *ZNF423* |
| *ALG13* | *BBS7* | *CEP164* | *EP300* | *GATA3* | *INPP5E* | *MYO1E* | *PDE6D* | *SIX1* | *TMEM231* | *WDPCP* |  |
| *ALMS1* | *BBS10* | *CEP290* | *EYA1* | *GDNF* | *INVS* | *NEK1* | *PKD1* | *SIX2* | *TMEM237* | *WDR19* |  |
| *ANKS3* | *BBS12* | *CHD1L* | *FAT3* | *GLIS2* | *IQCB1* | *NEK8* | *PKD2* | *SIX5* | *TNXB* | *WDR34* |  |
| *ANKS6* | *BMP2* | *CHD4* | *FAT4* | *GREM1* | *ITGA8* | *NODAL* | *PKHD1* | *SLIT2* | *TP53* | *WDR35* |  |
| *ANLN* | *BMP4* | *CHRM3* | *FGF2* | *GRIP1* | *KAL1* | *NPHP1* | *PLCE1* | *SOX17* | *TRAF3IP1* | *WDR60* |  |
| *APOL1* | *BMP7* | *CITED1* | *FGF9* | *HOXA13* | *KIAA0586* | *NPHP3* | *PODXL* | *SPRY1* | *TRAP1* | *WNT4* |  |
| *ARL6* | *C5orf42* | *COQ6* | *FGF20* | *HOXD11* | *KIF14* | *NPHP4* | *PTHB1* | *SRGAP1* | *TRIM32* | *WNT5A* |  |
| *ARL13B* | *CC2D2A* | *CSPP1* | *FGFR2* | *HNF1B* | *LAMB2* | *NPHS2* | *REN* | *TBC1D1* | *TRPC6* | *WNT7A* |  |

Gene list of SureSelect version 5

| *ACE* | *BBS1* | *CEP41* | *DYNC2H1* | *GANAB* | *IFT80* | *LMNA* | *PDE6D* | *SIX1* | *TMEM107* | *WDPCP* |
| --- | --- | --- | --- | --- | --- | --- | --- | --- | --- | --- |
| *AGT* | *BBS2* | *CEP83* | *DZIP1L* | *GATA3* | *IFT81* | *LMX1B* | *PIBF1* | *SIX2* | *TMEM138* | *WDR19* |
| *AGTR1* | *BBS4* | *CEP104* | *EP300* | *GDNF* | *IFT122* | *LZTFL1* | *PKD1* | *SIX5* | *TMEM216* | *WDR34* |
| *AGTR2* | *BBS5* | *CEP120* | *EVC* | *GLIS2* | *IFT140* | *MKKS* | *PKD2* | *SLIT2* | *TMEM231* | *WDR35* |
| *AHI1* | *BBS7* | *CEP164* | *EVC2* | *GLIS3* | *IFT172* | *MKS1* | *PKHD1* | *SOX17* | *TMEM237* | *WDR60* |
| *ALG9* | *BBS10* | *CEP290* | *EXOC4* | *GRIP1* | *INPP5E* | *MUC1* | *PTHB1* | *SPRY1* | *TNXB* | *WNT4* |
| *ALMS1* | *BBS12* | *CHD1L* | *EXOC8* | *GRLF1* | *INVS* | *NEK1* | *REN* | *SRGAP1* | *TRAF3IP1* | *WT1* |
| *ANKS3* | *C2CD3* | *CHD4* | *EYA1* | *HNF1B* | *IQCB1* | *NEK8* | *RET* | *TBC1D1* | *TRIM32* | *XPNPEP3* |
| *ANKS6* | *C5orf42* | *CHRM3* | *FAN1* | *HOXA13* | *ITGA8* | *NPHP1* | *ROBO2* | *TBC1D32* | *TSC1* | *ZNF423* |
| *ARL6* | *C21orf2* | *CITED1* | *FGF9* | *HPRT1* | *JAG1* | *NPHP3* | *RPGLIP1L* | *TBX1* | *TSC2* |  |
| *ARL13B* | *CC2D2A* | *CSPP1* | *FGF20* | *HYLS1* | *KAL1* | *NPHP4* | *SALL1* | *TBX18* | *TTBK2* |  |
| *ATXN10* | *CCDC28B* | *CTDNEP1* | *FGFR2* | *ICK* | *KIAA0586* | *OFD1* | *SARS2* | *TCTN1* | *TTC8* |  |
| *B9D1* | *CDC5L* | *DCDC2* | *FRAS1* | *IFN2* | *KIF7* | *PAX2* | *SCLT1* | *TCTN2* | *TTC21B* |  |
| *B9D2* | *CENPF* | *DDX59* | *FREM1* | *IFT27* | *KIF14* | *PAX8* | *SDCCAG8* | *TCTN3* | *UMOD* |  |
| *BBIP1* | *CEP19* | *DSTYK* | *FREM2* | *IFT43* | *LIFR* | *PBX1* | *SEC61A1* | *TMEM67* | *VANGL2* |  |

Gene list of SureSelect version 6

| *ACE* | *BBS1* | *CEP41* | *DYNC2H1* | *GANAB* | *IFT80* | *LMNA* | *PDE6D* | *SIX1* | *TMEM107* | *WDPCP* |
| --- | --- | --- | --- | --- | --- | --- | --- | --- | --- | --- |
| *AGT* | *BBS2* | *CEP83* | *DZIP1L* | *GATA3* | *IFT81* | *LMX1B* | *PIBF1* | *SIX2* | *TMEM138* | *WDR19* |
| *AGTR1* | *BBS4* | *CEP104* | *EP300* | *GDNF* | *IFT122* | *LZTFL1* | *PKD1* | *SIX5* | *TMEM216* | *WDR34* |
| *AGTR2* | *BBS5* | *CEP120* | *EVC* | *GLIS2* | *IFT140* | *MKKS* | *PKD2* | *SLIT2* | *TMEM231* | *WDR35* |
| *AHI1* | *BBS7* | *CEP164* | *EVC2* | *GLIS3* | *IFT172* | *MKS1* | *PKHD1* | *SOX17* | *TMEM237* | *WDR60* |
| *ALG9* | *BBS10* | *CEP290* | *EXOC4* | *GRIP1* | *INPP5E* | *MUC1* | *PTHB1* | *SPRY1* | *TNXB* | *WNT4* |
| *ALMS1* | *BBS12* | *CHD1L* | *EXOC8* | *GRLF1* | *INVS* | *NEK1* | *REN* | *SRGAP1* | *TRAF3IP1* | *WT1* |
| *ANKS3* | *C2CD3* | *CHD4* | *EYA1* | *HNF1B* | *IQCB1* | *NEK8* | *RET* | *TBC1D1* | *TRIM32* | *XPNPEP3* |
| *ANKS6* | *C5orf42* | *CHRM3* | *FAN1* | *HOXA13* | *ITGA8* | *NPHP1* | *ROBO2* | *TBC1D32* | *TSC1* | *ZNF423* |
| *ARL6* | *C21orf2* | *CITED1* | *FGF9* | *HPRT1* | *JAG1* | *NPHP3* | *RPGLIP1L* | *TBX1* | *TSC2* |  |
| *ARL13B* | *CC2D2A* | *CSPP1* | *FGF20* | *HYLS1* | *KAL1* | *NPHP4* | *SALL1* | *TBX18* | *TTBK2* |  |
| *ATXN10* | *CCDC28B* | *CTDNEP1* | *FGFR2* | *ICK* | *KIAA0586* | *OFD1* | *SARS2* | *TCTN1* | *TTC8* |  |
| *B9D1* | *CDC5L* | *DCDC2* | *FRAS1* | *IFN2* | *KIF7* | *PAX2* | *SCLT1* | *TCTN2* | *TTC21B* |  |
| *B9D2* | *CENPF* | *DDX59* | *FREM1* | *IFT27* | *KIF14* | *PAX8* | *SDCCAG8* | *TCTN3* | *UMOD* |  |
| *BBIP1* | *CEP19* | *DSTYK* | *FREM2* | *IFT43* | *LIFR* | *PBX1* | *SEC61A1* | *TMEM67* | *VANGL2* |  |

Gene list of SureSelect version 7

| *ACE* | *BBS1* | *CEP83* | *EP300* | *GFRA1* | *IFT80* | *LRIG2* | *PIBF1* | *SOX11* | *TNXB* |
| --- | --- | --- | --- | --- | --- | --- | --- | --- | --- |
| *ACTG2* | *BBS2* | *CEP104* | *EVC* | *GLIS2* | *IFT81* | *LRP5* | *PKD1* | *SOX17* | *TRAF3IP1* |
| *AGT* | *BBS4* | *CEP120* | *EVC2* | *GLIS3* | *IFT122* | *LZTFL1* | *PKD2* | *SPRY1* | *TRIM32* |
| *AGTR1* | *BBS5* | *CEP164* | *EXOC4* | *GPC3* | *IFT140* | *MAPKBP1* | *PKHD1* | *SRGAP1* | *TSC1* |
| *AGTR2* | *BBS7* | *CEP290* | *EXOC8* | *GREB1L* | *IFT172* | *MKKS* | *REN* | *SUFU* | *TSC2* |
| *AHI1* | *BBS9* | *CHD1L* | *EYA1* | *GREM1* | *INPP5E* | *MKS1* | *RET* | *TBC1D32* | *TTC8* |
| *ALG8* | *BBS10* | *CHD4* | *FAN1* | *GRIP1* | *INTU* | *MUC1* | *ROBO2* | *TBX1* | *TTC21B* |
| *ALG9* | *BBS12* | *CHD7* | *FGF20* | *HNF1B* | *INVS* | *NEK1* | *RPGRIP1L* | *TBX18* | *UMOD* |
| *ALMS1* | *BICC1* | *CHRM3* | *FGFR1* | *HOXA13* | *IQCB1* | *NEK8* | *SALL1* | *TCTEX1D2* | *UPK3A* |
| *ANKS6* | *C2CD3* | *CITED1* | *FGFR2* | *HPRT1* | *ITGA8* | *NOTCH2* | *SARS2* | *TCTN1* | *VANGL2* |
| *ARL3* | *C5orf42* | *CRB2* | *FRAS1* | *HPSE2* | *JAG1* | *NPHP1* | *SCLT1* | *TCTN2* | *WDPCP* |
| *ARL6* | *C8orf37* | *CSPP1* | *FREM1* | *HYLS1* | *KAL1* | *NPHP3* | *SDCCAG8* | *TCTN3* | *WDR19* |
| *ARL13B* | *CC2D2A* | *DCDC2* | *FREM2* | *INF2* | *KIAA0556* | *NPHP4* | *SEC61A1* | *TMEM67* | *WDR34* |
| *ARMC9* | *CCDC28B* | *DDX59* | *GANAB* | *IFT27* | *KIAA0586* | *OFD1* | *SIX1* | *TMEM107* | *WDR35* |
| *ATXN10* | *CDC5L* | *DNAJB11* | *GATA3* | *IFT43* | *KIAA0753* | *PAX2* | *SIX2* | *TMEM138* | *WDR60* |
| *B9D1* | *CDKN1C* | *DSTYK* | *GDF11* | *IFT52* | *KIF7* | *PAX8* | *SIX5* | *TMEM216* | *WNT4* |
| *B9D2* | *CENPF* | *DYNC2H1* | *GDNF* | *IFT57* | *KIF14* | *PBX1* | *SLIT2* | *TMEM231* | *WT1* |
| *BBIP1* | *CEP41* | *DZIP1L* | *GEN1* | *IFT74* | *LMX1B* | *PDE6D* | *SOX9* | *TMEM237* | *XPNPEP3* |
|  |  |  |  |  |  |  |  |  | *ZNF423* |

Gene list of SureSelect version 8

| *ACE* | *ATXN10* | *C8orf37* | *CHRM3* | *EYA1* | *GLIS3* | *IFT74* | *KIAA0753* | *NPHP3* | *SALL1* | *SUFU* | *TRAF3IP1* | *WT1* |
| --- | --- | --- | --- | --- | --- | --- | --- | --- | --- | --- | --- | --- |
| *ACTG2* | *B9D1* | *CC2D2A* | *CITED1* | *FAN1* | *GPC3* | *IFT80* | *KIF7* | *NPHP4* | *SARS2* | *TBC1D32* | *TRIM32* | *XPNPEP3* |
| *ADAMTS9* | *B9D2* | *CCDC28B* | *CRB2* | *FGF20* | *GREB1L* | *IFT81* | *KIF14* | *OFD1* | *SCLT1* | *TBX1* | *TSC1* | *ZNF423* |
| *AGT* | *BBIP1* | *CDC5L* | *CSPP1* | *FGFR1* | *GREM1* | *IFT122* | *LMX1B* | *PAX2* | *SDCCAG8* | *TBX18* | *TSC2* |  |
| *AGTR1* | *BBS1* | *CDKN1C* | *DCDC2* | *FGFR2* | *GRIP1* | *IFT140* | *LRIG2* | *PAX8* | *SEC61A1* | *TCTEX1D2* | *TTC8* |  |
| *AGTR2* | *BBS2* | *CENPF* | *DDX59* | *FRAS1* | *HNF1B* | *IFT172* | *LRP5* | *PBX1* | *SIX1* | *TCTN1* | *TTC21B* |  |
| *AHI1* | *BBS4* | *CEP41* | *DNAJB11* | *FREM1* | *HOXA13* | *INPP5E* | *LZTFL1* | *PDE6D* | *SIX2* | *TCTN2* | *UMOD* |  |
| *ALG8* | *BBS5* | *CEP83* | *DSTYK* | *FREM2* | *HPRT1* | *INTU* | *MAPKBP1* | *PIBF1* | *SIX5* | *TCTN3* | *UPK3A* |  |
| *ALG9* | *BBS7* | *CEP104* | *DYNC2H1* | *GANAB* | *HPSE2* | *INVS* | *MKKS* | *PKD1* | *SLIT2* | *TMEM67* | *VANGL2* |  |
| *ALMS1* | *BBS9* | *CEP120* | *DZIP1L* | *GATA3* | *HYLS1* | *IQCB1* | *MKS1* | *PKD2* | *SON* | *TMEM107* | *WDPCP* |  |
| *ANKS6* | *BBS10* | *CEP164* | *EP300* | *GDF11* | *INF2* | *ITGA8* | *MUC1* | *PKHD1* | *SOX9* | *TMEM138* | *WDR19* |  |
| *ARL3* | *BBS12* | *CEP290* | *EVC* | *GDNF* | *IFT27* | *JAG1* | *NEK1* | *REN* | *SOX11* | *TMEM216* | *WDR34* |  |
| *ARL6* | *BICC1* | *CHD1L* | *EVC2* | *GEN1* | *IFT43* | *KAL1* | *NEK8* | *RET* | *SOX17* | *TMEM231* | *WDR35* |  |
| *ARL13B* | *C2CD3* | *CHD4* | *EXOC4* | *GFRA1* | *IFT52* | *KIAA0556* | *NOTCH2* | *ROBO2* | *SPRY1* | *TMEM237* | *WDR60* |  |
| *ARMC9* | *C5orf42* | *CHD7* | *EXOC8* | *GLIS2* | *IFT57* | *KIAA0586* | *NPHP1* | *RPGRIP1L* | *SRGAP1* | *TNXB* | *WNT4* |  |

Gene list of SureSelect version 9

| *ACE* | *BBS2* | *CEP164* | *EVC2* | *GPC3* | *IFT172* | *MKS1* | *RET* | *TBC1D32* | *TTC21B* |
| --- | --- | --- | --- | --- | --- | --- | --- | --- | --- |
| *ACTG2* | *BBS4* | *CEP290* | *EXOC4* | *GREB1L* | *INPP5E* | *MUC1* | *ROBO2* | *TBX1* | *UMOD* |
| *ADAMTS9* | *BBS5* | *CHD1L* | *EXOC8* | *GREM1* | *INTU* | *NEK1* | *RPGRIP1L* | *TBX6* | *UPK3A* |
| *AGT* | *BBS7* | *CHD4* | *EYA1* | *GRIP1* | *INVS* | *NEK8* | *SALL1* | *TBX18* | *VANGL2* |
| *AGTR1* | *BBS9* | *CHD7* | *FAM149B1* | *HNF1B* | *IQCB1* | *NOTCH2* | *SARS2* | *TCTEX1D2* | *WDPCP* |
| *AGTR2* | *BBS10* | *CHRM3* | *FAN1* | *HOXA11* | *ITGA8* | *NPHP1* | *SCLT1* | *TCTN1* | *WDR19* |
| *AHI1* | *BBS12* | *CHRNA3* | *FGF20* | *HOXA13* | *JAG1* | *NPHP3* | *SDCCAG8* | *TCTN2* | *WDR34* |
| *ALG8* | *BICC1* | *CLCN5* | *FGFR1* | *HPRT1* | *KAL1* | *NPHP4* | *SEC61A1* | *TCTN3* | *WDR35* |
| *ALG9* | *C2CD3* | *CITED1* | *FGFR2* | *HPSE2* | *KIAA0556* | *NRIP1* | *SIX1* | *TMEM67* | *WDR60* |
| *ALMS1* | *C5orf42* | *CRB2* | *FRAS1* | *HYLS1* | *KIAA0586* | *OCRL* | *SIX2* | *TMEM107* | *WNT4* |
| *ANKS6* | *C8orf37* | *CSPP1* | *FREM1* | *INF2* | *KIAA0753* | *OFD1* | *SIX5* | *TMEM138* | *WT1* |
| *ARL3* | *CC2D2A* | *DCDC2* | *FREM2* | *IFT27* | *KIF7* | *PAX2* | *SLC20A1* | *TMEM216* | *XPNPEP3* |
| *ARL6* | *CCDC28B* | *DDX59* | *GANAB* | *IFT43* | *KIF14* | *PAX8* | *SLIT2* | *TMEM231* | *ZNF423* |
| *ARL13B* | *CDC5L* | *DNAJB11* | *GATA3* | *IFT52* | *LMX1B* | *PBX1* | *SON* | *TMEM237* |  |
| *ARMC9* | *CDKN1C* | *DSTYK* | *GDF11* | *IFT57* | *LRIG2* | *PDE6D* | *SOX9* | *TNXB* |  |
| *ATXN10* | *CENPF* | *DYNC2H1* | *GDNF* | *IFT74* | *LRP5* | *PIBF1* | *SOX11* | *TRAF3IP1* |  |
| *B9D1* | *CEP41* | *DYNC2LI1* | *GEN1* | *IFT80* | *LZTFL1* | *PKD1* | *SOX17* | *TRIM32* |  |
| *B9D2* | *CEP83* | *DZIP1L* | *GFRA1* | *IFT81* | *MAFB* | *PKD2* | *SPRY1* | *TSC1* |  |
| *BBIP1* | *CEP104* | *EP300* | *GLIS2* | *IFT122* | *MAPKBP1* | *PKHD1* | *SRGAP1* | *TSC2* |  |
| *BBS1* | *CEP120* | *EVC* | *GLIS3* | *IFT140* | *MKKS* | *REN* | *SUFU* | *TTC8* |  |

Gene list of SureSelect version 10

| *ACE* | *B9D2* | *CDC5L* | *CRB2* | *FAN1* | *GRIP1* | *IFT140* | *LRIG2* | *NPHP4* | *SALL1* | *TCTN2* | *UMOD* |
| --- | --- | --- | --- | --- | --- | --- | --- | --- | --- | --- | --- |
| *ACTG2* | *BBIP1* | *CDKN1C* | *CSPP1* | *FGF20* | *HNF1B* | *IFT172* | *LRP5* | *NRIP1* | *SARS2* | *TCTN3* | *UPK3A* |
| *AGT* | *BBS1* | *CENPF* | *DCDC2* | *FGFR1* | *HOXA11* | *INPP5E* | *LZTFL1* | *OCRL* | *SCLT1* | *TMEM67* | *VANGL2* |
| *AGTR1* | *BBS2* | *CEP41* | *DDX59* | *FGFR2* | *HOXA13* | *INTU* | *MAFB* | *OFD1* | *SDCCAG8* | *TMEM107* | *WDPCP* |
| *AGTR2* | *BBS4* | *CEP83* | *DNAJB11* | *FRAS1* | *HPRT1* | *INVS* | *MAPKBP1* | *PAX2* | *SEC61A1* | *TMEM138* | *WDR19* |
| *AHI1* | *BBS5* | *CEP104* | *DSTYK* | *FREM1* | *HPSE2* | *IQCB1* | *MKKS* | *PAX8* | *SIX1* | *TMEM216* | *WDR34* |
| *ALG8* | *BBS7* | *CEP120* | *DYNC2H1* | *FREM2* | *HYLS1* | *ITGA8* | *MKS1* | *PBX1* | *SIX2* | *TMEM231* | *WDR35* |
| *ALG9* | *BBS9* | *CEP164* | *DYNC2LI1* | *GANAB* | *INF2* | *JAG1* | *MUC1* | *PDE6D* | *SIX5* | *TMEM237* | *WDR60* |
| *ALMS1* | *BBS10* | *CEP290* | *DZIP1L* | *GATA3* | *IFT27* | *KAL1* | *MYH11* | *PIBF1* | *SON* | *TNXB* | *WNT4* |
| *ANKS6* | *BBS12* | *CFAP418* | *EP300* | *GDF11* | *IFT43* | *KIAA0556* | *MYL9* | *PKD1* | *SOX9* | *TOGARAM1* | *WT1* |
| *ARL3* | *BICC1* | *CHD1L* | *EVC* | *GDNF* | *IFT52* | *KIAA0586* | *MYLK* | *PKD2* | *SOX17* | *TRAF3IP1* | *XPNPEP3* |
| *ARL6* | *C2CD3* | *CHD4* | *EVC2* | *GFRA1* | *IFT57* | *KIAA0753* | *NEK1* | *PKHD1* | *SUFU* | *TRIM32* | *ZNF423* |
| *ARL13B* | *C5orf42* | *CHD7* | *EXOC4* | *GLIS2* | *IFT74* | *KIF7* | *NEK8* | *REN* | *TBX1* | *TSC1* |  |
| *ARMC9* | *CBWD1* | *CHRM3* | *EXOC8* | *GLIS3* | *IFT80* | *KIF14* | *NOTCH2* | *RET* | *TBX18* | *TSC2* |  |
| *ATXN10* | *CC2D2A* | *CHRNA3* | *EYA1* | *GPC3* | *IFT81* | *LMOD1* | *NPHP1* | *ROBO2* | *TCTEX1D2* | *TTC8* |  |
| *B9D1* | *CCDC28B* | *CLCN5* | *FAM149B1* | *GREB1L* | *IFT122* | *LMX1B* | *NPHP3* | *RPGRIP1L* | *TCTN1* | *TTC21B* |  |

Gene list of SureSelect version 11

| *ACE* | *BBIP1* | *CENPF* | *DDX59* | *FGFR2* | *HOXA13* | *INTU* | *MAFB* | *OFD1* | *SDCCAG8* | *TMEM138* | *VANGL2* |
| --- | --- | --- | --- | --- | --- | --- | --- | --- | --- | --- | --- |
| *ACTG2* | *BBS1* | *CEP41* | *DNAJB11* | *FRAS1* | *HPRT1* | *INVS* | *MAPKBP1* | *PAX2* | *SEC61A1* | *TMEM216* | *WDPCP* |
| *AGT* | *BBS2* | *CEP83* | *DSTYK* | *FREM1* | *HPSE2* | *IQCB1* | *MKKS* | *PAX8* | *SIX1* | *TMEM218* | *WDR19* |
| *AGTR1* | *BBS4* | *CEP104* | *DYNC2H1* | *FREM2* | *HYLS1* | *ITGA8* | *MKS1* | *PBX1* | *SIX2* | *TMEM231* | *WDR34* |
| *AGTR2* | *BBS5* | *CEP120* | *DYNC2LI1* | *GANAB* | *INF2* | *JAG1* | *MUC1* | *PDE6D* | *SIX5* | *TMEM237* | *WDR35* |
| *AHI1* | *BBS7* | *CEP164* | *DZIP1L* | *GATA3* | *IFT27* | *KAL1* | *MYH11* | *PIBF1* | *SOX9* | *TNXB* | *WDR60* |
| *ALG8* | *BBS9* | *CEP290* | *EP300* | *GDF11* | *IFT43* | *KIAA0556* | *MYL9* | *PKD1* | *SOX17* | *TOGARAM1* | *WNT4* |
| *ALG9* | *BBS10* | *CFAP418* | *EVC* | *GDNF* | *IFT52* | *KIAA0586* | *MYLK* | *PKD2* | *SUFU* | *TRAF3IP1* | *WT1* |
| *ALMS1* | *BBS12* | *CHD4* | *EVC2* | *GFRA1* | *IFT57* | *KIAA0753* | *NEK1* | *PKHD1* | *TBX1* | *TRIM32* | *XPNPEP3* |
| *ANKS6* | *BICC1* | *CHD7* | *EXOC4* | *GLIS2* | *IFT74* | *KIF7* | *NEK8* | *REN* | *TBX18* | *TSC1* | *ZNF423* |
| *ARL3* | *C2CD3* | *CHRM3* | *EXOC8* | *GLIS3* | *IFT80* | *KIF14* | *NOTCH2* | *RET* | *TCTEX1D2* | *TSC2* |  |
| *ARL6* | *C5orf42* | *CHRNA3* | *EYA1* | *GPC3* | *IFT81* | *LMOD1* | *NPHP1* | *ROBO2* | *TCTN1* | *TTC8* |  |
| *ARL13B* | *CBWD1* | *CLCN5* | *FAM149B1* | *GREB1L* | *IFT122* | *LMX1B* | *NPHP3* | *RPGRIP1L* | *TCTN2* | *TTC21B* |  |
| *ARMC9* | *CC2D2A* | *CRB2* | *FAN1* | *GRIP1* | *IFT140* | *LRIG2* | *NPHP4* | *SALL1* | *TCTN3* | *TXNDC15* |  |
| *B9D1* | *CCDC28B* | *CSPP1* | *FGF20* | *HNF1B* | *IFT172* | *LRP5* | *NRIP1* | *SARS2* | *TMEM67* | *UMOD* |  |
| *B9D2* | *CDKN1C* | *DCDC2* | *FGFR1* | *HOXA11* | *INPP5E* | *LZTFL1* | *OCRL* | *SCLT1* | *TMEM107* | *UPK3A* |  |

Gene list of SureSelect version 12

| *ACE* | *BBIP1* | *CEP41* | *DYNC2H1* | *GATA3* | *IFT57* | *LMOD1* | *NPHP4* | *SARS2* | *TMEM216* | *WDR34* |
| --- | --- | --- | --- | --- | --- | --- | --- | --- | --- | --- |
| *ACTG2* | *BBS1* | *CEP83* | *DYNC2LI1* | *GDF11* | *IFT74* | *LMX1B* | *NRIP1* | *SCLT1* | *TMEM218* | *WDR35* |
| *AGT* | *BBS2* | *CEP104* | *DZIP1L* | *GDNF* | *IFT80* | *LRIG2* | *OCRL* | *SDCCAG8* | *TMEM231* | *WDR60* |
| *AGTR1* | *BBS4* | *CEP120* | *EP300* | *GFRA1* | *IFT81* | *LRP5* | *OFD1* | *SEC61A1* | *TMEM237* | *WNT4* |
| *AGTR2* | *BBS5* | *CEP164* | *EVC* | *GLIS2* | *IFT122* | *LZTFL1* | *PAX2* | *SIX1* | *TNXB* | *WT1* |
| *AHI1* | *BBS7* | *CEP290* | *EVC2* | *GPC3* | *IFT140* | *MAFB* | *PAX8* | *SIX2* | *TOGARAM1* | *XPNPEP3* |
| *ALG5* | *BBS9* | *CHD4* | *EXOC4* | *GREB1L* | *IFT172* | *MAPKBP1* | *PBX1* | *SIX5* | *TRAF3IP1* | *ZNF423* |
| *ALG8* | *BBS10* | *CHD7* | *EXOC8* | *GRIP1* | *INPP5E* | *MKKS* | *PDE6D* | *SOX17* | *TRIM32* |  |
| *ALG9* | *BBS12* | *CHRM3* | *EYA1* | *HNF1B* | *INTU* | *MKS1* | *PIBF1* | *SUFU* | *TSC1* |  |
| *ALMS1* | *BICC1* | *CHRNA3* | *FAM149B1* | *HOXA11* | *INVS* | *MUC1* | *PKD1* | *TBX1* | *TSC2* |  |
| *ANKS6* | *C2CD3* | *CLCN5* | *FAN1* | *HOXA13* | *IQCB1* | *MYH11* | *PKD2* | *TBX18* | *TTC8* |  |
| *ANOS1* | *C5orf42* | *CRB2* | *FGF20* | *HPRT1* | *ITGA8* | *MYL9* | *PKHD1* | *TCTEX1D2* | *TTC21B* |  |
| *ARL3* | *C8orf37* | *CSPP1* | *FGFR1* | *HPSE2* | *JAG1* | *MYLK* | *REN* | *TCTN1* | *TXNDC15* |  |
| *ARL6* | *CBWD1* | *DACT1* | *FGFR2* | *HYLS1* | *KIAA0556* | *NEK1* | *RET* | *TCTN2* | *UMOD* |  |
| *ARL13B* | *CC2D2A* | *DCDC2* | *FRAS1* | *INF2* | *KIAA0586* | *NEK8* | *ROBO1* | *TCTN3* | *UPK3A* |  |
| *ARMC9* | *CCDC28B* | *DDX59* | *FREM1* | *IFT27* | *KIAA0753* | *NOTCH2* | *ROBO2* | *TMEM67* | *VANGL2* |  |
| *B9D1* | *CDKN1C* | *DNAJB11* | *FREM2* | *IFT43* | *KIF7* | *NPHP1* | *RPGRIP1L* | *TMEM107* | *WDPCP* |  |
| *B9D2* | *CENPF* | *DSTYK* | *GANAB* | *IFT52* | *KIF14* | *NPHP3* | *SALL1* | *TMEM138* | *WDR19* |  |
