# Supplementary material for: Phenotype and genotype of autosomal dominant tubulointerstitial kidney disease in a Japanese cohort
Source: Clin Exp Nephrol. 2025 Feb 20;29(6):788–96. doi: 10.1007/s10157-025-02629-4 (PMC12125067; doi:10.1007/s10157-025-02629-4)
Supplement: Supplementary file 4 — Supplementary file4 (DOCX 16 KB) Supplementary Table 4: References of Reports in the study [file 10157_2025_2629_MOESM4_ESM.docx]

Supplementary Table 2: References of Reports in the study.

1. Tsuchida S, Hara Y, Hori C, Hiraoka M, Suzuki S, Mayumi M. Prominent medial hypertrophy of renal arterioles in an infant with hyporeninemic hypoaldosteronism. Pediatr Nephrol. 1999;13:230-2.

2. Okada E, Morisada N, Horinouchi T, Fujii H, Tsuji T, Miura M, Katori H, Kitagawa M, Morozumi K, Toriyama T, Nakamura Y, Nishikomori R, Nagai S, Kondo A, Aoto Y, Ishiko S, Rossanti R, Sakakibara N, Nagano C, Yamamura T, Ishimori S, Usui J, Yamagata K, Iijima K, Imasawa T, Nozu K. Detecting MUC1 Variants in Patients Clinicopathologically Diagnosed With Having Autosomal Dominant Tubulointerstitial Kidney Disease. Kidney Int Rep. 2022;7:857-66.

3. Kuma A, Tamura M, Ishimatsu N, Miyamoto T, Serino R, Ishimori S, Morisada N, Iijima K, Yamada S, Takeuchi M, Abe H, Otsuji Y. A novel UMOD gene mutation associated with uromodulin-associated kidney disease in a young woman with moderate kidney dysfunction. Intern Med. 2015;54:631-5.
